# Genome wide identification of novel DNA methylation driven prognostic markers in colorectal cancer

**DOI:** 10.1038/s41598-024-60351-9

**Published:** 2024-07-08

**Authors:** Yuhua Ma, Yuanxin Li, Zhahong Wen, Yining Lai, Kulaixijiang Kamila, Jing Gao, Wang-yang Xu, Chengxiang Gong, Feifan Chen, Liuqing Shi, Yunzhi Zhang, Hanzhang Chen, Min Zhu

**Affiliations:** 1 Xinjiang Key Laboratory of Clinical Genetic Testing and Biomedical Information, Karamay Central Hospital, Karamay, 834099 Xin Jiang China; 2 Department of Pathology, Karamay Central Hospital, No. 67, Junggar Road, Karamay, 834099 Xin Jiang China; 3 Department of Pathology, Zhabei Central Hospital of Shanghai, No. 619, Zhonghua New Road, Jing’an District, Shanghai, 200070 China; 4 Singlera Genomics (Shanghai) Ltd., Shanghai, 201318 China

**Keywords:** Methylation analysis, Prognostic markers, Colorectal cancer

## Abstract

Colorectal cancer (CRC) stands as a major contributor to cancer-related fatalities within China. There is an urgent need to identify accurate biomarkers for recurrence predicting in CRC. Reduced representation bisulfite sequencing was used to perform a comparative analysis of methylation profiles in tissue samples from 30 recurrence to 30 non-recurrence patients with CRC. Least absolute shrinkage and selection operator method was performed to select the differential methylation regions (DMRs) and built a DNA methylation classifier for predicting recurrence. Based on the identified top DMRs, a methylation classifier was built and consisted of eight hypermethylated DMRs in CRC. The DNA methylation classifier showed high accuracy for predicting recurrence with an area under the receiver operator characteristic curve of 0.825 (95% CI 0.680–0.970). The Kaplan–Meier survival analysis demonstrated that CRC patients with high methylation risk score, evaluated by the DNA methylation classifier, had poorer survival than low risk score (Hazard Ratio 4.349; 95% CI 1.783–10.61, P = 0.002). And only CRC patients with low methylation risk score could acquire benefit from adjuvant therapy. The DNA methylation classifier has been proved as crucial biomarkers for predicting recurrence and exhibited promising prognostic value after curative surgery in patients with CRC.

## Introduction

Colorectal cancer (CRC) ranks as the third most prevalent cancer globally and continues to be the leading cause of cancer-related fatalities^1–3^. The incidence and mortality rates of CRC have been on the rise in recent years, attributed to factors such as unhealthy diets and environmental pollution. At the time of diagnosis, a large proportion of patients with colon adenocarcinoma already have advanced-stage disease^4^. CRC is a highly heterogeneous disease^5^, and survival rates following surgery vary greatly among patients at different stages. For example, while the 5-year survival rate for stage IV CRC patients is below 10%, stage I patients exhibit survival rates exceeding 90%^6^. The substantial individual differences in prognosis among CRC patients pose a huge challenge due to the disease's inherent heterogeneity^7^. Currently, carcinoembryonic antigen (CEA) is the primary biomarker used for surveillance in CRC patients^8^. However, the sensitivity and specificity of CEA still require improvement^9^. Therefore, the development of effective biomarkers is essential to enhance the clinical outcomes of CRC patients.

Epigenetic diversity in CRC has garnered increasing attention in recent years. Extensive studies have been conducted to unravel the DNA methylation landscape of CRC^10,11^. Aberrant DNA methylation leads to alterations in epigenetic modifications closely associated with tumor initiation and progression^12–14^. Methylation patterns can serve as the earliest detectable neoplastic signal during tumorigenesis^15–17^. The methylation status in gene promoters or other regulatory elements is referred to as the cytosine-phosphate-guanine (CpG) island methylation phenotype (CIMP). CIMP hypermethylation in CRC is associated with clinical characteristics such as female gender, advanced age, right-sided colon tumors, microsatellite instability-high (MSI-H) status, and *BRAF* mutations^18^. Aberrant DNA methylation of specific genes regulates the expression of methylation-driven genes in CRC^19^.

Colorectal cancer is a complex disease involving genes among intricate signaling pathways that contribute to its onset and progression^20^. The methylation status of gene promoters, including *MGMT*, *MLH1*, *APC1A*, *SHOX2*, *RASSF1A*, and *PHD1*, is closely related to the occurrence and progression of colon adenocarcinoma^21–23^. A DNA methylation classifier consisting of eight differentially methylated sites (DMCs) demonstrated promising prognostic value in stage II CRC. The high-risk group stratified by this methylation classifier exhibited poor survival outcomes^24^. ColonAiQ assay was a circulating tumor DNA methylation method and identified CRC in 86% while maintaining a specificity of 92%^25^. Postoperative findings at month 1 revealed that patients testing positive through ColonAiQ were 17.5 times more likely to experience a relapse compared to those testing negative^26^. Although these studies have reported that methylation feature of certain genes can predict recurrence of colorectal cancer, the performance of the detection is limited.

The objective of this study was to perform genome-wide DNA methylation analysis to identify more effective biomarker that could identify the high-risk recurrence of CRC patients. A cohort of thirty CRC patients with recurrence and thirty patients without recurrence was enrolled for the study. We performed a comprehensive analysis of the genome using reduced representation bisulfite sequencing (RRBS) assay to identify specific differential methylation regions (DMRs) associated with CRC recurrence. We built a DNA methylation classifier and evaluated the performance of predicting recurrence and determining the prognostic value in CRC.

## Materials and methods

### Patient enrollment and sample collection

In this study, a total of 30 CRC patients who experienced postoperative recurrence and 30 CRC patients without recurrence within three years after surgery were enrolled from 2015 to 2020 at Karamay Central Hospital in Xinjiang, China. Detailed clinical information, including age, gender, TNM stage, and pathological type, was collected for each patient. None of the patients had received neoadjuvant therapy prior to surgery. Written informed consent was obtained from all patients before sample collection. Tumor tissue samples were obtained during the surgical procedure and preserved through paraffin embedding.

### DNA extraction and methylation sequencing

According to manufacturer's instructions, formalin-fixed paraffin-embedded (FFPE) samples from CRC patients were subjected to DNA extraction with the QIAamp DNA FFPE tissue Kit (Qiagen, Hilden, Germany). Quality control measures were implemented to ensure the integrity of the extracted DNA samples. The methylation feature of CpG sites were assessed by RRBS method, performed as previously described^27^. DNA input between 50 and 100 ng was subjected to digestion with MspI enzyme prior to ligation with a methylated adaptor containing complementary sticky ends. Subsequently, the ligation products underwent bisulfite conversion using the Methylcode Bisulfite Conversion Kit (ThermoFisher, MECOV50), followed by purification and recovery steps. To introduce a barcode for Illumina sequencing, the converted DNA was amplified. The libraries were then sequenced on the Illumina Hiseq X10 platform.

### Immunohistochemical Detection of mismatch repair proteins

A Roche Bench MarkXT automated immunohistochemistry instrument was applied, using EnVision Two-step method, primary antibody (MLH1, PMS2, MSH2 and MSH6) and secondary antibody were purchased from Roche Biotechnology Development Co. Interpretation criteria: MLH1, PMS2, MSH2 and MSH6 positive signals were located in the nucleus, positive staining tumor and surrounding mesenchymal tissue nuclei were brownish, negative staining tumor nuclei were not colored, but normal epithelial or mesenchymal tissue nuclei around the tumor were brownish.

### DNA methylation sequencing data analysis

The Illumina bcl2fastq software was performed to do the demultiplexing of reads (https://support.illumina.com/sequencing/sequencing_software/bcl2fastq-conversion-software.html). FASTQ data were adapter-trimmed by the first 2 bases from each end with trim-galore (https://www.bioinformatics.babraham.ac.uk/projects/trim_galore). Single-end reads were generated by merging the paired-end read FASTQ files. The single-end reads were aligned to the bisulfite-converted human reference genome (version hg19) using Bismark (https://www.bioinformatics.babraham.ac.uk/projects/bismark/)^28^ and Bowtie v.1 (http://bowtie-bio.sourceforge.net/bowtie2/index.shtml)^29^, resulting in BAM files. The mapped bam files were subsequently utilized for further analysis. CpG methyRate calculating was performed with Bismark to identify the DMCs between recurrence and nonrecurrence CRC samples. A minimum of five CpG sites was required for one DMR. The final DMC was determined based on the following standard: |methylation difference|≥ 12% and q value < 0.01. Similarly, the final DMR was determined with the following standard: q value < 0.05.

### Gene Ontology and KEGG functional pathway enrichment analysis

To elucidate the functional implications of the DMRs in CRC progression, DAVID database was employed for conducting Gene Ontology (GO) and KEGG pathway analyses (available at [https://david.ncifcrf.gov/])^30,31^. This analysis aimed to identify top GO categories and enriched KEGG pathways associated with CRC based on the DMRs. Statistical significance was determined using a threshold of P < 0.05.

### Statistical analysis

Statistical analyses were carried out using R version 4.3.2 (https://cran.r-project.org/) and GraphPad Prism version 9.0 (https://www.graphpad.com/). To compare the differences in methylation levels between recurrence and non-recurrence CRC samples, both Student's t-test and non-parametric Mann–Whitney test were employed, according to the distribution of the data. Receiver operator characteristic (ROC) analysis was employed to assess the performance of DMRs and the DNA methylation classifier in identifying patients with a high risk of recurrence. This analysis was conducted using the timeROC function, utilizing 5-year survival data for assessment. The Cox proportional hazard model was utilized to assess the associations between methylation features and patient recurrence. Least absolute shrinkage and selection operator (LASSO) was performed to select the DMRs for recurrence predicting by cross validation in R software. Survival curve analysis was performed using the Kaplan–Meier method, and the significance of differences was assessed using the log-rank test. Disease-free survival (DFS) was defined as the duration from the surgery date to either recurrence or the last follow-up. P < 0.05 was considered statistically significant.

### Ethics approval and consent to participate

The studies involving human participants were reviewed and approved by Karamay Central Hospital Ethics committee. All methods were performed following the relevant guidelines and regulations. The Ethics Number: YL-2020-25. All patients enrolled had provided written informed consent.

## Results

### Patient characteristics

The study enrolled a total of 60 CRC patients who did not receive neoadjuvant therapy. Table 1 summarizes the clinical and pathological characteristics of the patients. All patients underwent radical R0 resection. The median age of the cohort was 67.5 years (range 40–84), with 48.3% of the patients being female. The median follow-up time for recurrence and non-recurrence CRC patients was 28.6 months and 63.4 months, respectively.

**Table 1:** Clinicopathologic characteristics of colorectal cancer patients.

Characteristics	Cohort (n = 60)
Age (Median ± SD, years)	67.5 ± 11.7
Gender	
Female	29 (48.3%)
Male	31 (51.7%)
Tumor location	
Colon	26 (43.3%)
Rectum	34 (56.7%)
Lymphatic invasion	
Yes	30 (50%)
No	28 (46.7%)
Unknown	2 (3.3%)
Venous invasion	
Yes	23 (38.3%)
No	33 (55.0%)
Unknown	4 (6.7%)
Peripheral nerve invasion	
Yes	28 (46.7%)
No	28 (46.7%)
Unknown	4 (6.7%)
Stage	
I	8 (13.3%)
II	21 (35.0%)
III	28 (46.7%)
IV	1 (1.7%)
Unknown	2 (3.3%)
MMR status	
dMMR	6 (10.0%)
pMMR	54 (90.0%)
Recurrence	
Yes	30 (50%)
No	30 (50%)

MMR, mismatch repair; dMMR, deficient mismatch repair; pMMR, mismatch repair proficient.

### Identification of recurrence associated methylation feature

The study employed the RRBS assay to perform genome-wide methylation sequencing in 30 recurrence and 30 non-recurrence CRC patients. A total of 17,918 DMCs and 12,119 DMRs were identified between recurrence and non-recurrence samples. DMRs were determined using the following criteria: q-value < 0.05 (Supplementary Fig. S1A). The distribution of DMRs was investigated, revealing that 46.07% were distributed in promoters and 10.51% in exons (Supplementary Fig. S1B). 6857 DMRs located in gene promoters and exons were selected for further analysis to identify the top DMRs.

### GO and pathway items contributing to CRC recurrence

Based on the DMRs distributed in promoters and exons, DMRs associated genes were analyzed for GO analysis and pathway enrichment. Thorough annotation and analysis of genes were conducted, focusing on their established molecular functions. The analysis revealed prominent enrichment in molecular function categories, notably including ATP binding, calcium ion binding and serine/threonine/tyrosine kinase (Supplementary Fig. S2A). The top 21 most enriched pathways were displayed, including cAMP signaling pathway, renin secretion, gap junctions, and Circadian entrainment, and so on (Supplementary Fig. S2B).

### Identification of DMRs independently associated with recurrence

**Figure 1:**
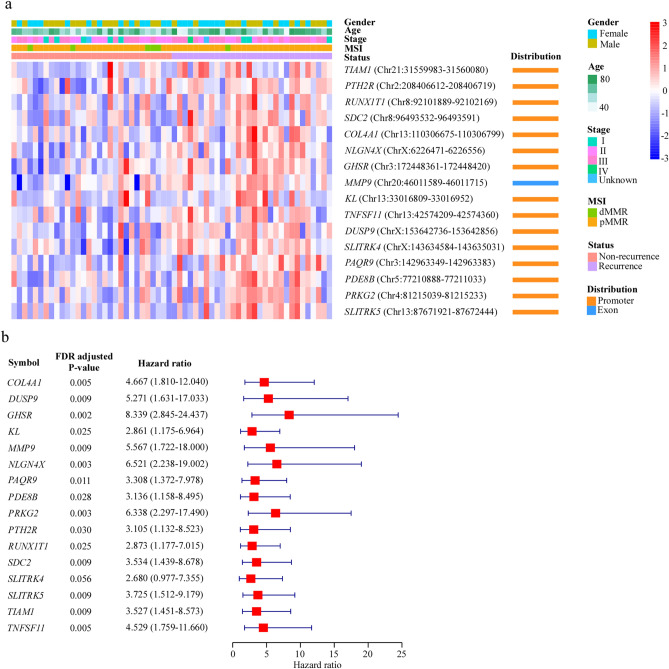
Selected top DNA methylation markers identified between recurrence and non-recurrence CRC patients**.** (**a**) Cluster analysis heatmap for annotated genes associated with the 16 selected DMRs in CRC. Red shows hypermethylation and blue shows hypomethylation. (**b**) The univariate Cox regression analysis conducted on selected DMR genes in CRC. The hazard ratio (HR) of recurrence is presented as solid boxes, and the 95% confidence interval (CI) is indicated using the open-ended horizontal lines.

Using the differential methylation levels of all DMRs, the Cox proportional hazard model analysis was performed. The involvement of these DMR genes in the top 21 enriched pathways was analyzed. The Cox proportional hazard model analysis is followed on the standard: *p* value < 0.05 and hazard ratio (HR) > 2. This analysis identified 16 DMRs which altered DMR methylation levels were significantly associated with recurrence in CRC, respectively (Supplementary Table S1). The unsupervised hierarchical clustering result indicated that these 16 DMRs demonstrated significant differences between recurrence and non-recurrence CRC patients (Fig. 1a). The Cox proportional hazard model analysis showed that each one of 16 DMRs was closely associated with CRC recurrence (Fig. 1b and Supplementary Table S2).

### Constructing and assessing the DNA methylation classifier for CRC recurrence predicting

**Figure 2:**
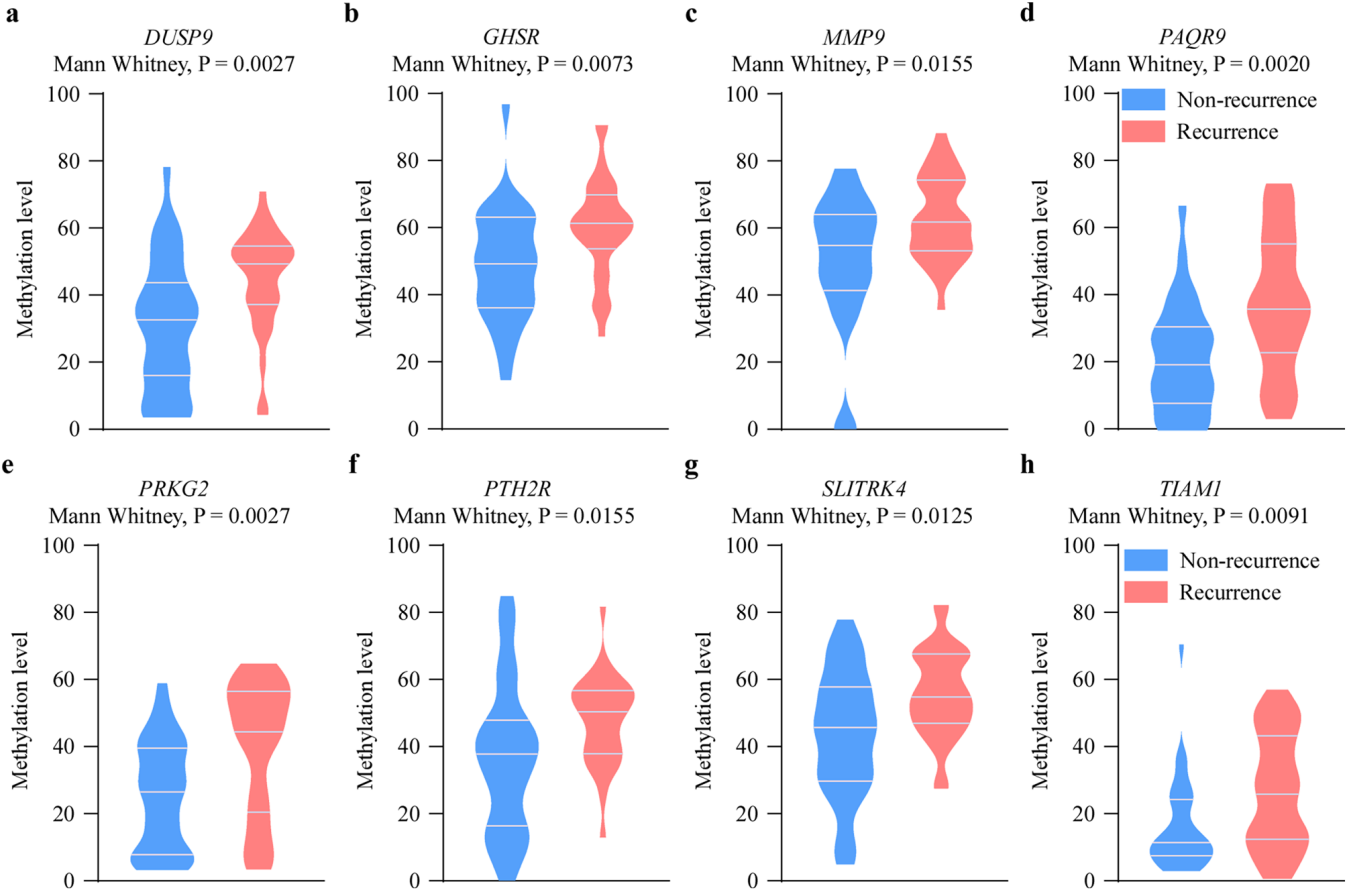
DMRs show close association with recurrence in colorectal cancer. *β* value of 8 DMR biomarkers of *DUSP9* (**a**), *GHSR* (**b**), *MMP9* (**c**), *PAQR9* (**d**), *PRKG2* (**e**), *PTH2R* (**f**), *SLITRK4* (**g**) and *TIAM1* (**h**) in the methylation feature model was compared between recurrence (n = 30) and non-recurrence (n = 30) CRC samples.

Our objective was to develop a CRC recurrence predicting classifier to separate recurrence CRC patients from the non-recurrence patients. To accomplish this, the cohort of 60 CRC patients was randomly divided into two groups: a training set and a validation set, utilizing a 2:1 ratio. The performance of recurrence prediction among 16 individual DMRs was assessed and compared using survival-based ROC curve analysis in the training set (Supplementary Fig. S3 and Supplementary Table S2). Using the training samples, eight most discriminatory DMRs for CRC recurrence were identified by threefold cross-validation Lasso-Cox regression model. We chose the best choice of lambda (lambda.min = 0.0854) and found the contribution coefficient of 8 variables were compressed to zero (Supplementary Fig. S4A,B). The recurrence predicting DNA methylation classifier included Dual Specificity Phosphatase 9 (*DUSP9*), Growth Hormone Secretagogue Receptor (*GHSR*), Matrix Metallopeptidase 9 (*MMP9*), Progestin and AdipoQ Receptor Family Member 9 (*PAQR9*), Protein Kinase Cyclic Guanosine Monophosphate-Dependent 2 (*PRKG2*), Parathyroid Hormone 2 Receptor (*PTH2R*), SLIT And NTRK Like Family Member 4 (*SLITRK4*) and TIAM Rac1 Associated GEF 1 (*TIAM1*). The detailed chromosome sites of the eight DMRs were indicated in Supplementary Table S1. Except for *MMP9* DMR located in the exon, the DMRs of the remaining genes were located within the promoter region. And all eight DMR markers in the methylation classifier exhibited significant hypermethylation status in the recurrence samples (Fig. 2).

The methylation risk score was assessed as follow:

$${\text{Methylation risk score}}\,{ = }\,{0}{\text{.00935 * DUSP9}}\,{ + }\,{0}{\text{.00845 * GHSR}}\,{ + }\,{0}{\text{.00351 * MMP9}}\,{ + }\,{0}{\text{.00556 * PAQR9}}\,{ + }\,{0}{\text{.00841 * PRKG2}}\,{ + }\,{0}{\text{.00469 * PTH2R}}\,{ + }\,{0}{\text{.00474 * SLITRK4}}\,{ + }\,{0}{\text{.00730 * TIAM1}}{.}$$

**Figure 3:**
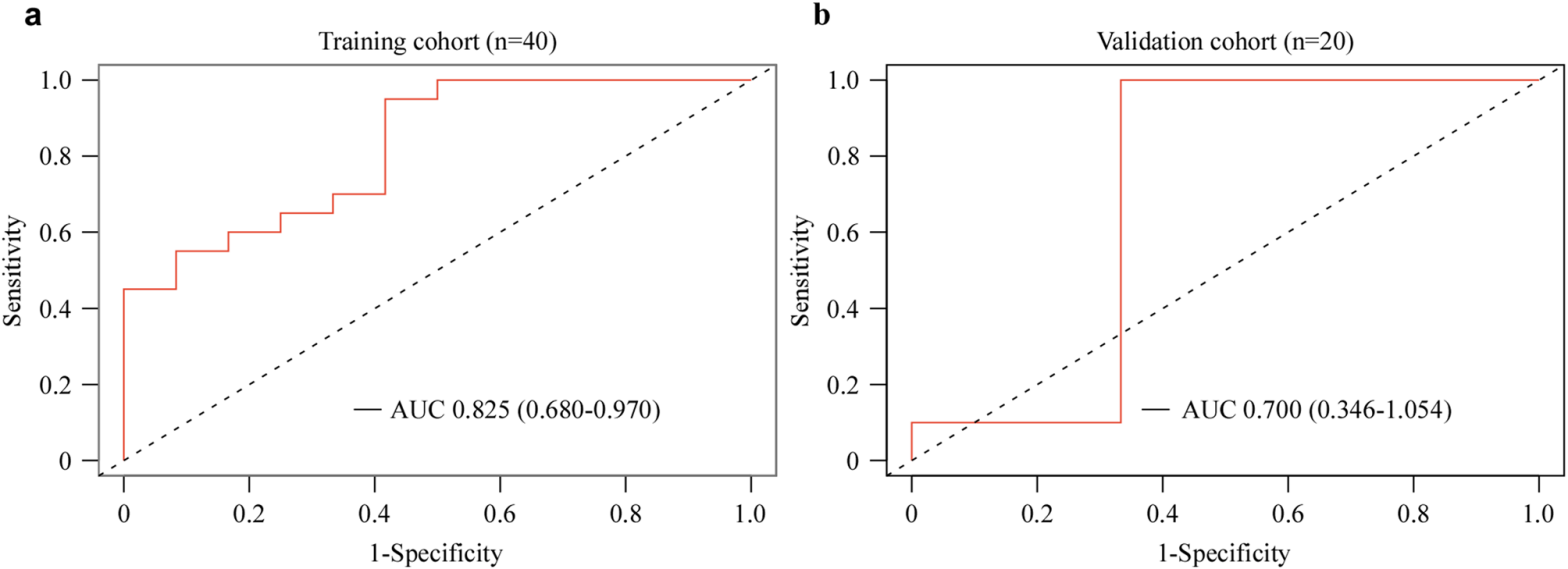
A methylation feature model built by using the 8 selected DMR markers accurately classified non-recurrence and recurrence CRC patients. (**a**) ROC curves representing the performance in the training cohort. (**b**) Displaying the ROC curves for the validation cohort.

The DNA methylation classifier achieved a high area under the roc curve (AUC) of 0.825 (95% CI 0.680–0.970) in the training set (Fig. 3a). The DNA methylation classifier underwent validation using the allocated validation set and showed an achieved AUC of 0.700 (95% CI 0.346–1.054), demonstrating its consistency and robustness (Fig. 3b).

### Prognosis prediction for CRC patients by the DNA methylation classifier

**Figure 4:**
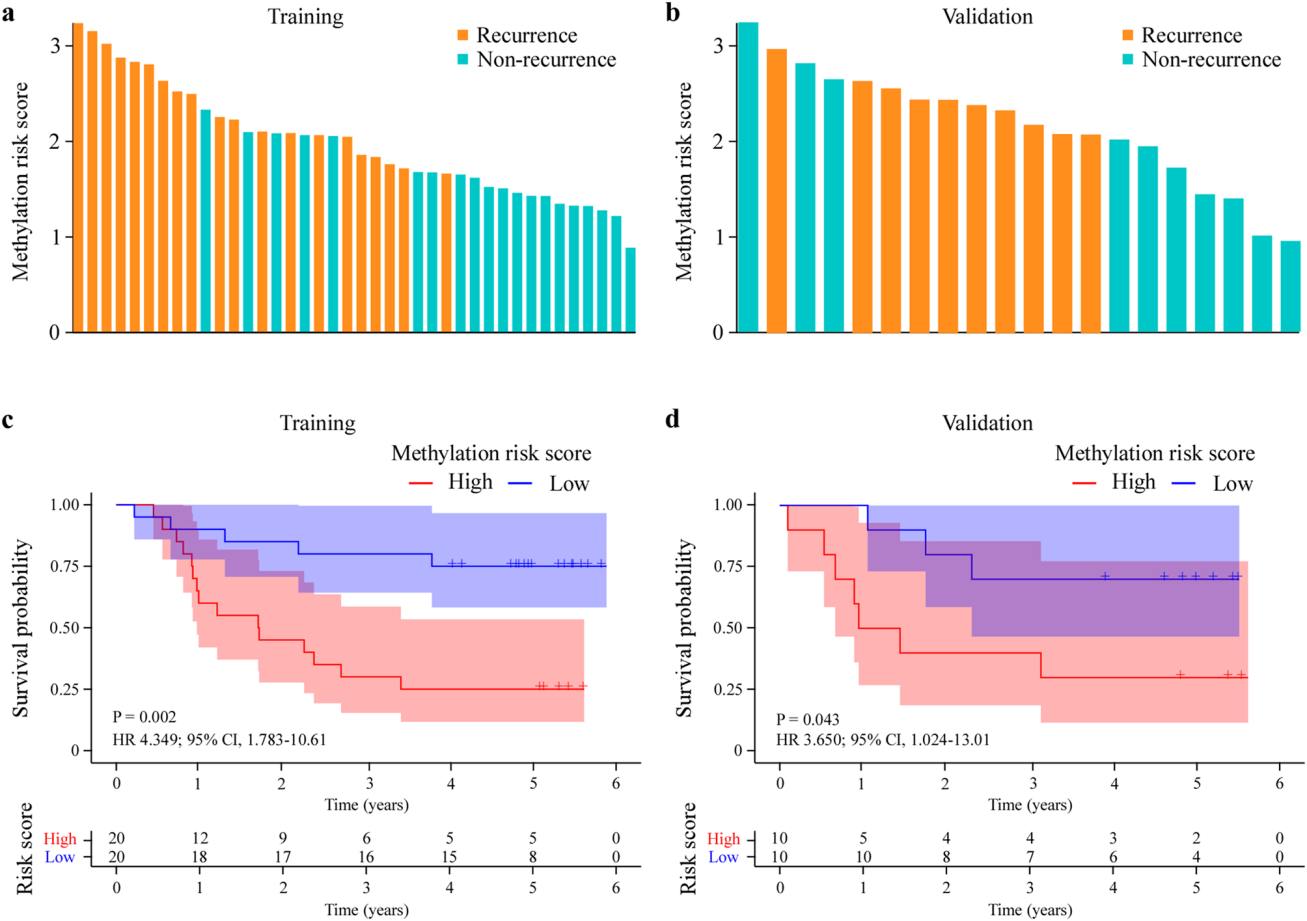
Prognosis value of the DNA methylation classifier. A waterfall plot displays the risk scores generated by the DNA methylation classifier in both the training (**a**) and validation cohort (**b**). DFS Kaplan–Meier survival of CRC patients predicted by DNA methylation classifier in the training (**c**) and validation cohort (**d**).

We conducted prognostic predictions for colorectal CRC patients by leveraging the DNA methylation risk score, which was calculated by the equation of methylation risk score. The distribution of the DNA methylation risk score was estimated in the training set and validation set. The result indicated that high methylation risk score was related closely to recurrence status, while low methylation risk score associated with non-recurrence status (Fig. 4a,b). To examine the prognosis value of the DNA methylation classifier, DFS Kaplan–Meier survival analysis was performed. The median of the methylation risk score served as the threshold to distinguish between the high-risk and low-risk cohorts. In the training set, the result of Kaplan–Meier survival analysis demonstrated that CRC patients with high methylation risk score had shooter DFS than patients with low-risk score (Hazard Ratio (HR) 4.349; 95% CI 1.783–10.61, P = 0.002) (Fig. 4c). The equivalent prognosis value of the DNA methylation classifier was then indicated in the validation set (HR 3.650; 95% CI 1.024–13.01, P = 0.043) (Fig. 4d). Multivariate Cox regression analysis showed that the DNA methylation classifier was an independent prognostic biomarker in colorectal cancer (Supplementary Figs. S5 and S6).

### Outcome prediction of adjuvant therapy for CRC patients by the DNA methylation classifier

**Figure 5:**
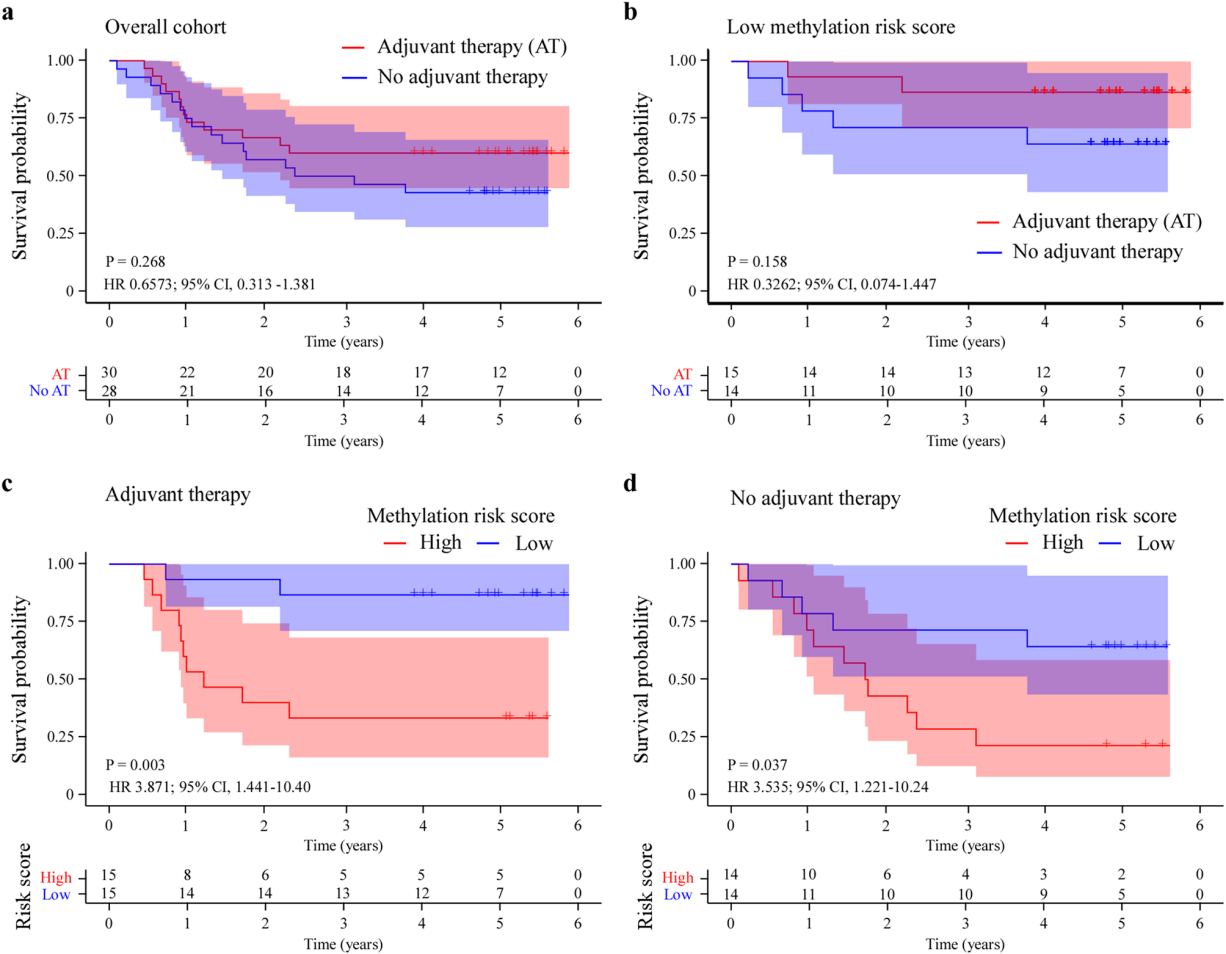
The Kaplan–Meier curves of DFS for CRC patients treated with adjuvant therapy. DFS Kaplan–Meier curves for CRC patients with adjuvant therapy and not with adjuvant therapy in overall cohort (**a**) and in low methylation risk score group (**b**). DFS Kaplan–Meier analysis of methylation risk high-score and low-score CRC patients in chemotherapy group (**c**) and non-chemotherapy group (**d**).

We investigated the impact of the DNA methylation classifier on treatment decisions by assessing the outcomes of adjuvant therapy. The DFS Kaplan–Meier survival result showed that CRC patients with adjuvant therapy had a better DFS than CRC patients without adjuvant therapy, even though the difference wasn’t significant (HR 0.6573; 95% CI 0.313–1.381, P = 0.268) (Fig. 5a). CRC patients with high methylation risk score had a poorer DFS than CRC patients with low methylation risk score, no matter treated with adjuvant therapy (HR 3.871; 95% CI 1.441–10.40, P = 0.003) or without adjuvant therapy (HR 3.535; 95% CI 1.221–10.24, P = 0.037) (Fig. 5c,d). In low methylation risk score group, CRC patients with adjuvant therapy treatment had a better DFS than patients without adjuvant therapy (HR 0.3262; 95% CI 0.074–1.447, P = 0.158) (Fig. 5b).

## Discussion

The current standard method for postoperative recurrence assessment in CRC patients is TNM staging, but it has limitations, especially for patients with stages II and III, where the recurrence may not correlate well with the stage. This study aimed to address this issue by enrolling 30 CRC patients with recurrence and 30 CRC patients without recurrence. Genome-wide screening of methylation sites was conducted to identify recurrence-associated DMRs. The study successfully built a DNA methylation classifier consisting of eight hypermethylated DMRs, which was closely related to disease recurrence in CRC. ROC curve analysis revealed the AUC of 0.825 and 0.700 for predicting CRC recurrence in the training and validation set, respectively. Survival analysis further demonstrated that the high methylation risk score group identified by the DNA methylation classifier had a significantly shorter DFS, no matter receiving treatment of adjuvant therapy or not. The CRC patients with the low methylation risk score could acquire the benefit from treatment of adjuvant therapy.

Epigenetic inheritance and modifications influence tumor occurrence and progression. DNA methylation appears early in carcinogenesis, so many clusters of methylation variants could be detectable^32^. A study identified eight CpG sites, which were closely associated with recurrence, including *EGFR*, *EFNB2*, *GNAL*, *PPAPDC1A* and *RGS12*. Prognosis model of these eight CpG sites resulted in AUCs for recurrence prediction of 0.75 and 0.71 in the training and validation sets, respectively^24^. Hypermethylation of the *BCAT1*/*IKZF1* predicted a high risk of recurrence in stage I-III CRC. Three-year recurrence free survival (RFS) in hypermethylation and hypomethylation was 56.5% and 83.3%, respectively^33^. A methylation model had a sensitivity of 87.5% and specificity of 94.12% in predicting disease recurrence in stage III CRC^34^. Research that performed data mining of TCGA database also found methylation marker genes associated with colorectal cancer recurrence^35–37^. These findings are also of great interest, but methylation biomarkers for recurrence predicting have not been investigated from a genome-wide scale in CRC.

In this study, we applied the genome-wide DNA methylation assay by RRBS sequencing. A DNA methylation classifier consisting of eight hypermethylated DMRs was built and evaluated, including *DUSP9*, *GHSR*, *MMP9*, *PAQR9*, *PRKG2*, *PTH2R*, *SLITRK4* and *TIAM1*. *GHSR* has been pinpointed as a promising early detection biomarker for a wide spectrum of cancers^38,39^. *PRKG2* emerges as a pivotal factor governing cell proliferation and apoptosis, notably in the context of tumorigenesis^40,41^. Fecal MMP-9 effectively identified CRC patients with a sensitivity of 89.3% and a specificity of 91.2%^42^. Reduced DUSP9 expression in CRC showed close correlation with poor survival rates^43^. Elevated nuclear TIAM1 levels in clinical specimens correlate with improved survival among CRC patients^44^. Our analysis showed that hypermethylation of genes in the DNA methylation classifier would be promising to play a crucial role in recurrence monitoring in CRC patients.

There are some limitations to the study. Firstly, the study enrolled fewer patients and was distributed from stages I to III. Secondly, the validation set's small size is insufficient for accurately evaluating the classifier's performance in CRC. Future studies should consider validating the result and its recurrence prediction performance in an independent validation set to strengthen the findings.

In conclusion, the study successfully identified recurrence-associated DMRs through genome-wide screening of methylation sites in CRC patients. The discovery of the eight DMRs-related genes within the classifier holds substantial promise for monitoring the recurrence of colorectal cancer. These findings have important implications for improving postoperative recurrence assessment in CRC patients, especially for those with stages II and III where traditional methods may be less accurate. By utilizing DNA methylation changes as biomarkers, clinicians may be able to better predict and manage recurrence in CRC patients, ultimately leading to improved outcomes and quality of life for these patients. Further research is needed to validate these findings and explore potential therapeutic interventions based on DNA methylation changes in CRC recurrence.

### Supplementary Information

Supplementary Information.

## Data Availability

The datasets analyzed during the current study are available from the corresponding authors on reasonable request.
