# Supplementary material for: Genome wide identification of novel DNA methylation driven prognostic markers in colorectal cancer
Source: Sci Rep. 2024 Jul 8;14:15654. doi: 10.1038/s41598-024-60351-9 (PMC11231291; doi:10.1038/s41598-024-60351-9)
Supplement: Supplementary file 1 — Supplementary Information. [file 41598_2024_60351_MOESM1_ESM.pdf]

Genome-wide identification of novel DNA methylation driven prognostic markers in colorectal cancer

Yuhua Ma[1,2†], Yuanxin Li[1,2†], Zhahong Wen[4†], Yining Lai[1,2], Kulaixijiang Kamila[1,2], Jing Gao[1,2], Wang-yang Xu[4], Chengxiang Gong[4], Feifan Chen[4], Liuqing Shi[4], Yunzhi Zhang[4], Hanzhang Chen[3*], Min Zhu[1,2*]

Supplementary Table 1. List of 16 top significantly differentially methylated regions between recurrence and non-recurrence CRC samples.

Symbol	Chr	Start	End	q value FDR	Mean methylation difference	TranscriptId
COL4A1	chr13	110306675	110306799	0.00000271	12.097195	ENST00000543140.6
DUSP9	chrX	153642736	153642856	1.02E-12	11.558832	ENST00000370167.8
GHSR	chr3	172448361	172448420	0.00010767	11.665557	ENST00000427970.1
KL	chr13	33016809	33016952	9.74E-15	11.194425	ENST00000487852.1
MMP9	chr20	46011589	46011715	7.53E-08	12.681703	ENST00000372330.3
NLGN4X	chrX	6226471	6226556	0.00000411	11.438688	ENST00000469740.1
PAQR9	chr3	142963349	142963383	3.72E-10	16.838851	ENST00000340634.5
PDE8B	chr5	77210888	77211033	8.89E-16	10.96797	ENST00000333194.8
PRKG2	chr4	81215039	81215233	7.89E+31	13.973284	ENST00000264399.5
PTH2R	chr2	208406612	208406719	9.24E-11	12.839237	ENST00000272847.6
RUNX1T1	chr8	92101889	92102169	1.33E-14	10.258555	ENST00000519577.5
SDC2	chr8	96493532	96493591	5.65E-11	13.384127	ENST00000520233.1
SLITRK4	chrX	143634584	143635031	1.28E-15	12.353495	ENST00000596188.2
SLITRK5	chr13	87671921	87672444	1.78E-16	13.789065	ENST00000325089.7
TIAM1	chr21	31559983	31560080	2.47E-09	10.177099	ENST00000469412.5
TNFSF11	chr13	42574209	42574360	9.35E-10	13.377602	ENST00000544862.5

Supplementary Table 2. Cox proportional hazard model analysis of 16 top DMRs in CRC.

Symbols	Chr.	Start	Stop	HR	Lower 0.95	Upper 0.95	*FDR adjusted P*-value	AUC
COL4A1	chr13	110306675	110306799	4.667	1.810	12.040	0.0054	0.633
DUSP9	chrX	153642736	153642856	5.271	1.631	17.033	0.0094	0.671
GHSR	chr3s	172448361	172448420	8.339	2.845	24.437	0.0016	0.538
KL	chr13	33016809	33016952	2.861	1.175	6.964	0.0254	0.527
MMP9	chr20	46011589	46011715	5.567	1.722	18.000	0.0094	0.675
NLGN4X	chrX	6226471	6226556	6.521	2.238	19.002	0.0032	0.683
PAQR9	chr3	142963349	142963383	3.308	1.372	7.978	0.0112	0.721
PDE8B	chr5	77210888	77211033	3.136	1.158	8.495	0.0281	0.679
PRKG2	chr4	81215039	81215233	6.338	2.297	17.490	0.0032	0.767
PTH2R	chr2	208406612	208406719	3.105	1.132	8.523	0.0296	0.688
RUNX1T1	chr8	92101889	92102169	2.873	1.177	7.015	0.0254	0.721
SDC2	chr8	96493532	96493591	3.534	1.439	8.678	0.0094	0.617
SLITRK4	chrX	143634584	143635031	2.680	0.977	7.355	0.0556	0.633
SLITRK5	chr13	87671921	87672444	3.725	1.512	9.179	0.0094	0.738
TIAM1	chr21	31559983	31560080	3.527	1.451	8.573	0.0094	0.742
TNFSF11	chr13	42574209	42574360	4.529	1.759	11.660	0.0054	0.621

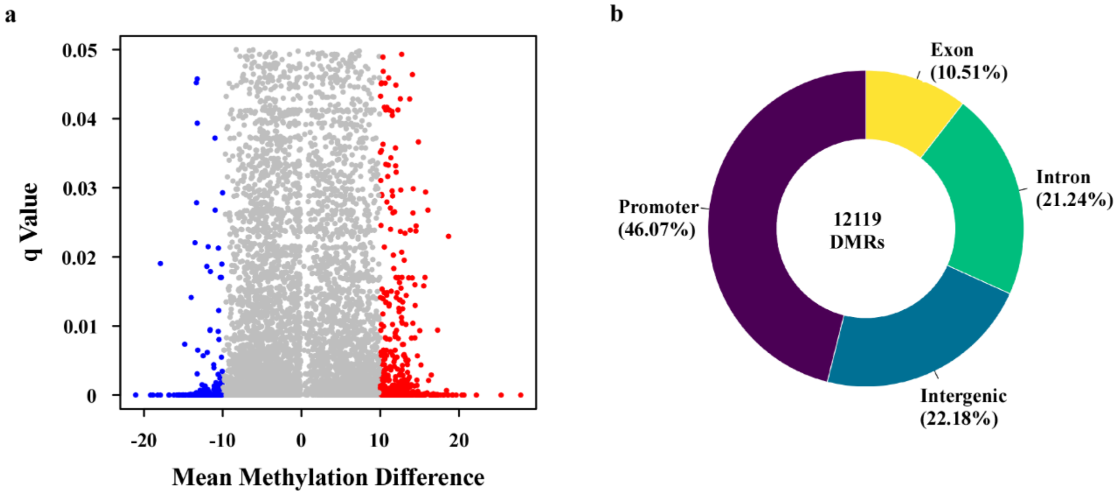

Supplementary Figure 1. Differentially methylated regions detected in tissue samples from recurrence and non-recurrence CRC patients. **a** Volcano plot illustrating results of DMRs analysis in CRC patients. The analysis criteria for DMRs: q value ≤ 0.05. **b** Distribution in genome of DMRs' locations.

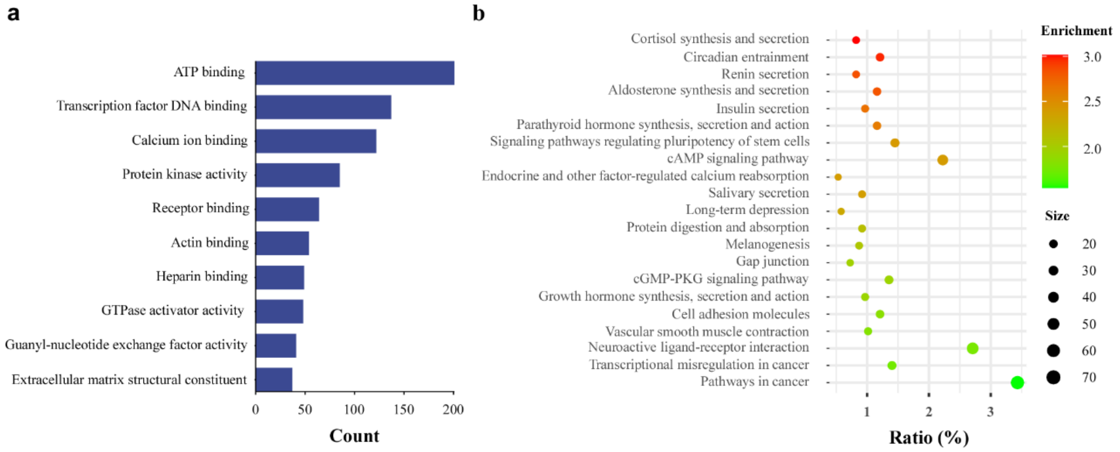

Supplementary Figure 2. Gene ontology and KEGG enrichment analysis of the DMR markers associated genes in CRC. **a** Top molecular function categories analysis of genes. **b** Top KEGG functional pathway enrichment analysis of genes.

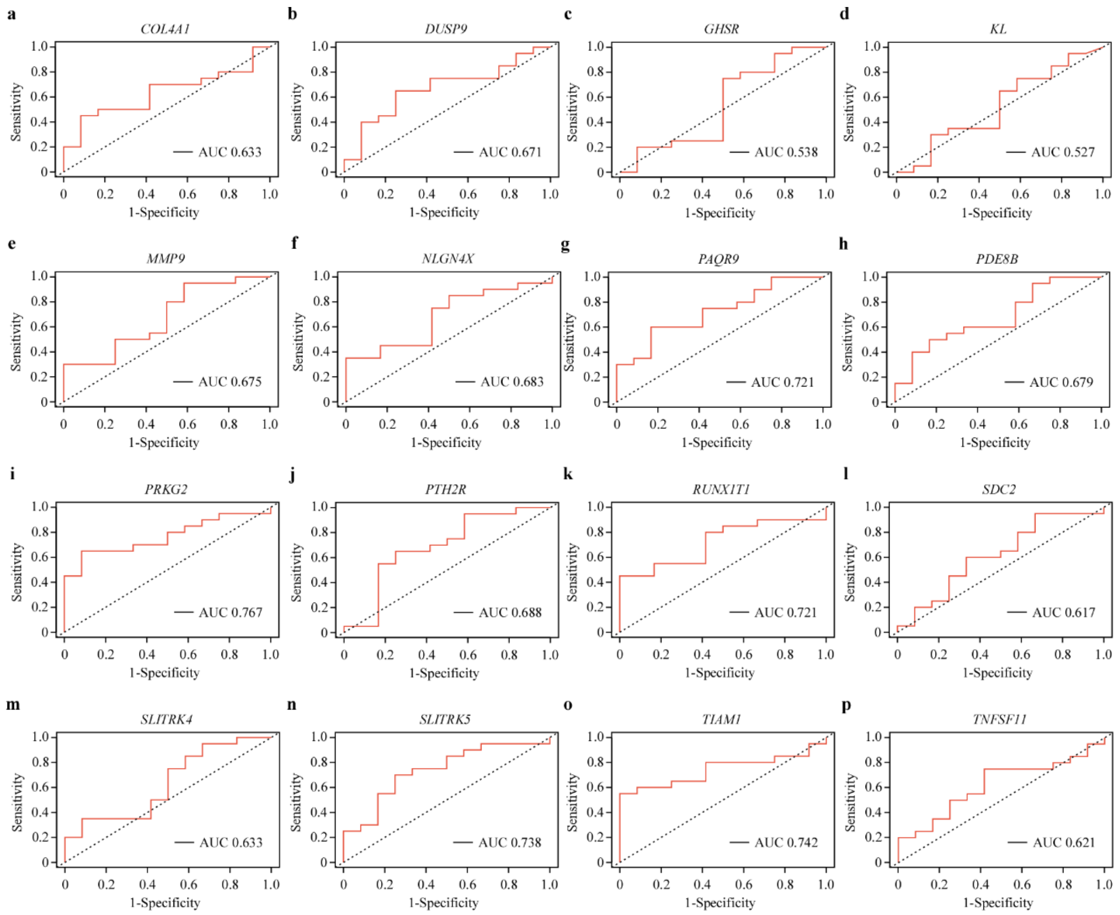

Supplementary Figure 3. ROC analysis for identifying CRC recurrence based on the mean β value of 16 DNA methylation regions. ROC curve indicates the performance of recurrence predicting of DMRs' associated genes, including *COL4A1* (**a**), *DUSP9* (**b**), *GHSR* (**c**), *KL* (**d**), *MMP9* (**e**), *NLGN4X* (**f**), *PAQR9* (**g**), *PDE8B* (**h**), *PRKG2* (**i**), *PTH2R* (**j**), *RUNX1T1* (**k**), *SDC2* (**l**), *SLITRK4* (**m**), *SLITRK5* (**n**), *TIAM1* (**o**) and *TNFSF11* (**p**). AUC: area under the curve.

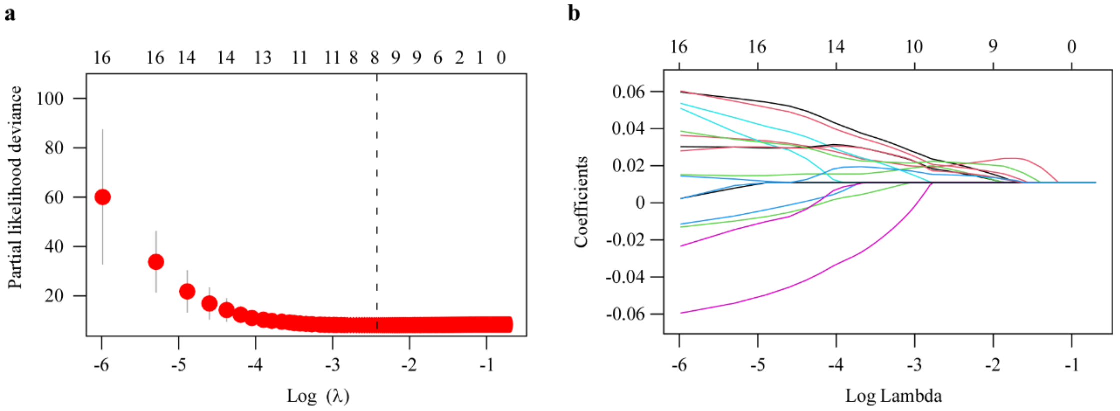

Supplementary Figure 4. Screening of variables based on Lasso-Cox regression model. (**a**) The selection process of the optimum value of the parameter λ in the Lasso-Cox regression model. (**b**) The variation characteristics of the coefficient of variables.

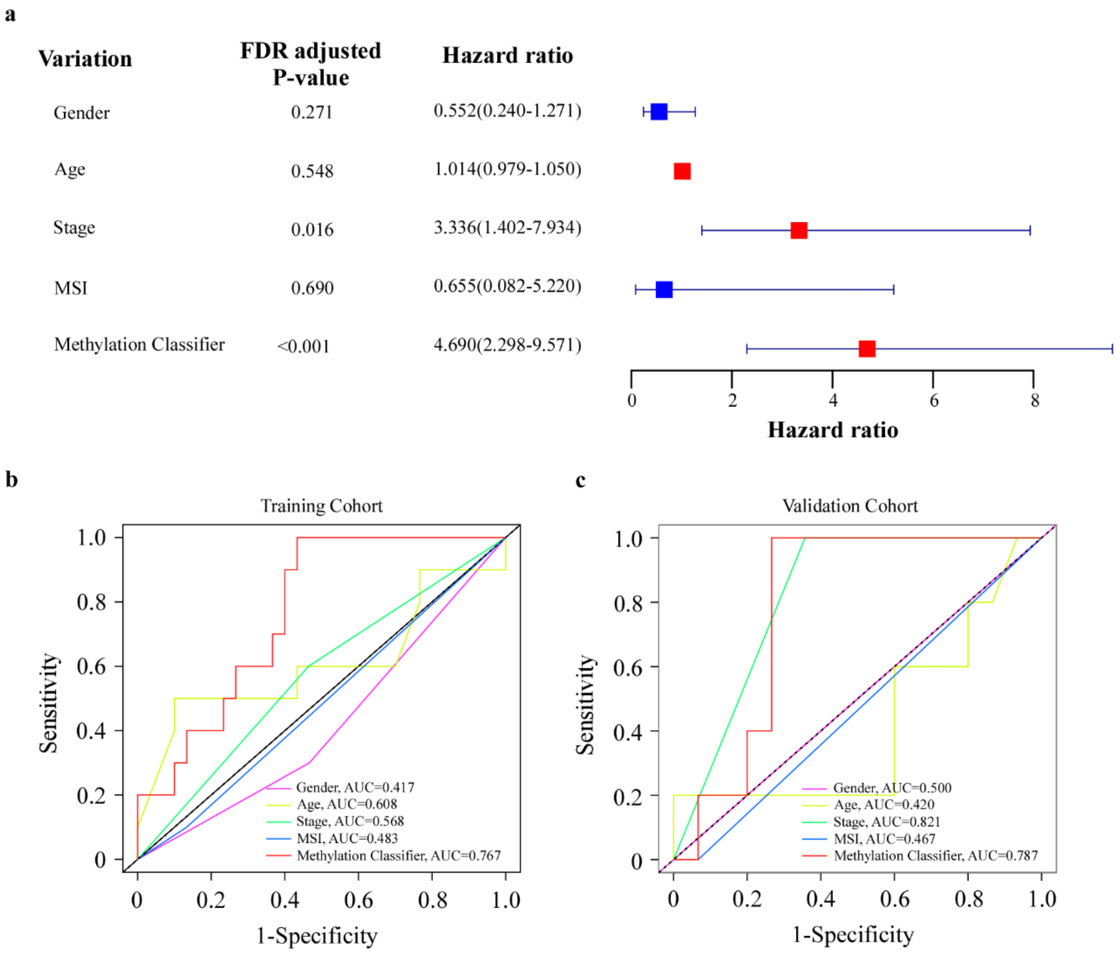

Supplementary Figure 5. DNA methylation classifier is an independent prognostic biomarker in colorectal cancer. **(a)** Multivariate Cox regression analysis of clinicopathologic factors and DNA methylation classifier. ROC graphs of the clinicopathologic factors and DNA methylation classifier on predicting recurrence in their corresponding training set **(b)** and validation set **(c)**.

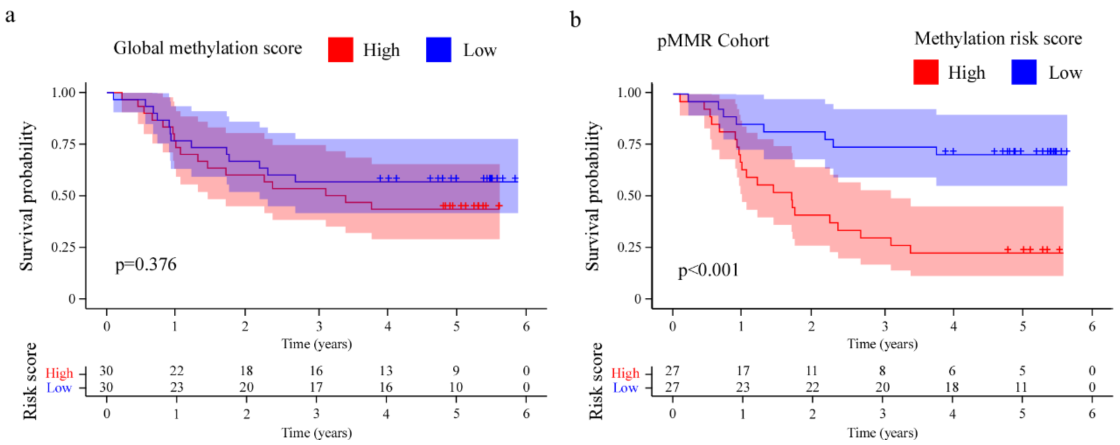

Supplementary Figure 6. Kaplan-Meier analysis of DFS between global methylation score-high and -low groups **(a)**, and of DFS predicted by the DNA methylation classifier consisting of selected eight DMRs in pMMR cohort **(b)**.
